# Hydrochloride fasudil attenuates brain injury in ICH rats

**DOI:** 10.1515/tnsci-2020-0100

**Published:** 2020-04-20

**Authors:** Limin Li, Xiaoli Lou, Kunlun Zhang, Fangping Yu, Yingchun Zhao, Ping Jiang

**Affiliations:** Department of Neurology, The Affiliated Shanghai Songjiang Central Hospital of Shanghai Jiao Tong University, Central Hospital of Shanghai Jiao Tong University School of Medicine, Shanghai, China

**Keywords:** intracerebral hemorrhage, hydrochloride fasudil, Rho-associated protein kinase, inflammation, brain injury

## Abstract

**Aim:**

The aim of this study was to investigate the neuroprotective effects of hydrochloride fasudil (HF) in rats following intracerebral hemorrhage (ICH).

**Methods:**

Male Wistar rats were randomly divided into four groups: normal, sham-operated, ICH, and ICH/HF. ICH was induced by injection of non-anticoagulant autologous arterial blood into the right caudate nucleus. The levels of Rho-associated protein kinase 2 (ROCK2) mRNA and protein around the site of the hematoma were measured by quantitative real-time polymerase chain reaction and enzyme-linked immunosorbent assay (ELISA), respectively. The levels of interleukin-6 and tumor necrosis factor-α in serum were detected by ELISA. The inflammatory cells and changes in the neuronal morphology around the hematoma were visualized using hematoxylin and eosin and Nissl staining. Brain edema was measured by comparing wet and dry brain weights.

**Results:**

Following ICH, the levels of ROCK2 were significantly increased from day 1 to day 7. The levels of ROCK2 were significantly lower in rats treated with HF than in controls. The levels of inflammatory cytokines and brain water content were significantly higher in rats treated with HF than in controls. Administration of HF significantly reduced the levels of inflammatory cytokines and brain water content from day 1 to day 7. In the acute phase of ICH, a large number of neutrophils infiltrated the perihematomal areas. In comparison with the ICH group, the ICH/HF group showed markedly fewer infiltrating neutrophils on day 1. Nissl staining showed that ICH caused neuronal death and loss of neurons in the perihematomal areas at all time points and that treatment with HF significantly attenuated neuronal loss.

**Conclusions:**

HF exerts neuroprotective effects in ICH rats by inhibiting the expression of ROCK2, reducing neutrophil infiltration and production of inflammatory cytokines, decreasing brain edema, and attenuating loss of neurons.

## Abbreviations

BBB: blood–brain barrier
ELISA: enzyme-linked immunosorbent assay
HF: hydrochloride fasudil
ICH: intracerebral hemorrhage
IL-6: interleukin-6
NLR: neutrophil-to-lymphocyte ratio
qRT-PCR: quantitative real-time polymerase chain reaction
ROCK: Rho-associated protein kinases
TNF-α: tumor necrosis factor-α

## 1 Introduction

Intracerebral hemorrhage (ICH) is an acute cerebrovascular disease, with high morbidity and mortality, for which there is currently no satisfactory treatment. Although the pathogenesis of ICH is not completely understood, recent research shows that secondary injury plays a critical role in neurological deterioration in patients with ICH [1]. Increasing evidence has shown that inflammatory injury plays a criticl role in ICH-induced secondary brain injury and is linked to brain edema, neuronal damage, cell apoptosis, and immune damage [2–4]. Inhibition of the inflammatory response, and consequent reduction of brain edema, may thus represent a novel therapeutic strategy for the treatment of ICH.

Rho-associated protein kinases (ROCK), which belong to the AGC family (protein kinases A, G, and C) of serine–threonine kinases and include two isoforms: ROCK1 (also known as ROKb and p160 ROCK) and ROCK2 (also known as ROKa and Rho kinase), regulate a variety of cell functions and play key roles in cell contraction, inflammation, vascular leakage, and maintenance of the blood–brain barrier (BBB) [5–8]. ROCK1 is expressed prominently in the lung, liver, spleen, kidney, and testis, and ROCK2 is expressed preferentially in brain and heart [5]. Yamashitaa et al. showed that ROCK2 immunoreactivity was clearly increased in the ischemic brain, whereas ROCK1 was not high in either the intact or the ischemic brain in mice. These results are consistent with a previous report that ROCK2 is expressed in brain, whereas ROCK1 exhibits its highest expression levels in non-neuronal tissues [9].

The Rho/ROCK pathway has been found to be involved in cardiovascular diseases, neurological diseases, and cancer, and inhibition of ROCK can be beneficial for the therapy of these related diseases [5]. Based on the information we have collected, ROCK inhibitors can be roughly divided into several groups: isoquinoline derivatives, indazole derivatives, urea derivatives, aminopyrimidine derivatives, and others. Among them, Y27632 and fasudil are the most representative ROCK inhibitors, and they have been extensively used in biological experiments. Besides, some potent ROCK inhibitors have been pushed into clinical trials, including Y39983/RKI983, SAR407899, and AMA0076. The nonselective ROCK inhibitor fasudil was first approved in 1995 in Japan for the treatment of cerebral vasospasm after subarachnoid hemorrhage; since then, ROCK inhibitors have been used to treat spinal cord injury [10], Alzheimer’s disease [11], stroke [12], and Parkinson’s disease [13]. There are, however, few reports describing the effects of ROCK inhibitors on ICH.

In this study, we investigated the neuroprotective effect of hydrochloride fasudil (HF) by determining its effects on inflammatory response, brain edema, and neuronal injury in an autologous blood-induced model of ICH in rats.

## 2 Materials and methods

### 2.1 Animals and treatment groups

Adult male Wistar rats (250 ± 20 g) were purchased from the Experimental Animal Center of the First People’s Hospital Affiliated to Shanghai Jiao Tong University (Shanghai, China). One hundred seventeen rats were randomly divided into four groups: normal group (*n* = 9), sham-operated group (*n* = 36), ICH group (*n* = 36), and ICH/HF group (*n* = 36). The rats were fasted for 12 h before the experiments but had free access to water.


**Ethical approval:** The research related to animal use has been complied with all the relevant national regulations and institutional policies for the care and use of animals.

### 2.2 Administration of saline and HF

Rats were used in this study, and they were divided into groups receiving intraperitoneal injection of either saline or HF (12 mg/Kg). Then, the intravenous injection was changed to intraperitoneal injection, which was 11.15–11.81 mg/kg/d (the ratio of intraperitoneal injection to intravenous dose was 1.18–1.25). HF (MedChemExpress, Newark, NJ, USA) was dissolved in 2 mL of 0.9% saline solution and administered intraperitoneally once a day (12 mg/kg/d), starting 12 h after the onset of ICH. Rats in the sham-operated and ICH groups received an equal volume of normal saline.

### 2.3 ICH model

In rats, ICH was induced by injection of non-anticoagulant autologous arterial blood into the right caudate nucleus, as previously described [14]. Briefly, the rats were anesthetized by intraperitoneal injection of 10% chloral hydrate (0.4 mL/100 g) and placed in a brain stereotactic apparatus (Huaibei Zhenghua Biological Instrument Equipment Co., Ltd, Huaibei, China). The right caudate nucleus (0.2 mm anterior to coronal, 3.0 mm lateral to the bregma, and 6.0 mm ventral) was positioned in accordance with the “Stereotaxic Atlas of Rat Brain” [15]. Non-anticoagulant autologous blood (100 μL) was collected from the right femoral artery of the rats using a microsyringe and immediately injected into the right caudate nucleus at a rate of 10 μL/min. The needle was left in place for 15–20 min and then slowly removed. The skull was sealed using medical bone wax to prevent blood reflux, and the incision was sutured. The sham-operated rats underwent the same surgical procedure, except that no blood was injected.

When the rats had recovered from the anesthetic, the behavioral abnormalities were assessed by a blinded observer using Zea Longa scores [16] (0: rat has no neurological deficit; 1: rat is unable to completely extend contralateral forelimb; 2: rat circles to left when walking; 3: rat tumbles to left when walking; and 4: rat is unconscious and unable to walk). Scores of 1–3 were used for modeling.

### 2.4 Serum interleukin-6 and tumor necrosis factor-α levels

On days 0, 1, 3, 7, and 14, the rats were anesthetized by intraperitoneal injection of 10% chloral hydrate (0.4 mL/100 g). After appropriate skin preparation and sterilization, the chest was opened and blood was collected from the right atrium. Serum was isolated using standard procedures, and the levels of interleukin (IL)-6 and tumor necrosis factor (TNF)-α were determined using a commercial enzyme-linked immunosorbent assay (ELISA) kit (Affymetrix Inc., Santa Clara, CA, USA), according to the manufacturer’s protocol.

### 2.5 Quantitative real-time polymerase chain reaction

The levels of ROCK2 mRNA were measured using quantitative real-time polymerase chain reaction (qRT-PCR) on days 0, 1, 3, 7, and 14 after induction of ICH. Total RNA was isolated from the homogenates of 100 mg tissue surrounding the hematoma (not including hematomas) using a total RNA extraction kit (Invitrogen, Carlsbad, CA, USA), according to the manufacturer’s instructions. First-strand cDNA was synthesized from RNA using a first-strand cDNA synthesis kit (Thermo Scientific, Foster City, CA, USA). PCR was carried out using Maxima SYBR Green qPCR Master Mix (Thermo Scientific, Waltham, MA, USA). The primers (ROCK2, 5ʹ-AGATGTGAAGCCCGATAA-3ʹ [forward primer] and 5ʹ-ACACCTACAGACCACCAAT-3ʹ [reverse primer]; glyceraldehyde 3-phosphate dehydrogenase [GAPDH], 5ʹ-ATGATTCTACCCACGGCAAG-3ʹ [forward primer] and 5ʹ-CTGGAAGATGGTGATGGGTT-3ʹ [reverse primer]) were purchased from the Beijing Genomics Institute (Beijing, China). ROCK2 mRNA levels were compared with those of control rats (value in control rats set as 1.0).

### 2.6 ROCK2 protein expression in the perihematomal region

Brain tissues around the lesion sites in right caudate nucleus of rats were removed and homogenized with 1 mL animal tissue active protein extraction kit (Shenggong, Shanghai, China). Total protein was determined using a bicinchoninic acid protein assay kit (Beyotime, Shanghai, China) and ROCK2 levels were determined using a commercially available ELISA kit (LifeSpan BioSciences, Inc., Seattle, Washington, USA). In each case, the assay kit was used according to the manufacturer’s protocol. ROCK2 protein levels were calculated as a percentage of total protein.

### 2.7 Assessment of cerebral edema

Brain water content was used to evaluate cerebral edema on days 0, 1, 3, 7, and 14. The rats were killed by decapitation, and their brains were excised. A coronal incision was made to evaluate hematoma formation, and two pieces of tissues (∼2 mm^2^) were removed from the area around the hematoma. The tissues were weighed immediately to obtain wet weights and again after drying at 100°C for 24 h to obtain dry weights. The brain water content (%) in the perihematomal areas was calculated as follows:

([\text{wet}\hspace{.25em}\text{weight}\hspace{.25em}-\text{dry
                        weight}]/\text{wet}\hspace{.25em}\text{weight})\times 100 \%

### 2.8 Hematoxylin and eosin staining

The rats were exsanguinated via the right atrium and perfused with saline (200 mL) and then with 4% paraformaldehyde (200 mL) to fix the brain tissues *in vivo*. The rats were decapitated, and the brain tissues were fixed by plunging into 10% formaldehyde solution. The tissues were then dehydrated and embedded in paraffin and the sections (4 μm) were prepared for hematoxylin and eosin (H&E) staining. Hematoma was observed in the basal ganglia on days 1, 3, 7, and 14 after the onset of ICH. Inflammatory infiltration into perihematomal tissue was observed using a light microscope, and the data were analyzed using Motic Digital Medical Image software (Motic China Group Co., Ltd, Xiamen, China). Neutrophils were counted in four independent fields of view (×400).

### 2.9 Nissl staining

For Nissl staining, the brain tissues were fixed in 10% formaldehyde solution, dehydrated, embedded in paraffin using standard procedures, and cut into 4 μm sections using a rotary microtome (Leica, Solms, Hessen, Germany). The paraffin sections were dewaxed and rehydrated, stained using toluidine blue solution (0.5%), differentiated in 95% alcohol, dried, and cleared using xylene. Morphological changes in the neurons were observed using a light microscope, and the data were analyzed using Motic Digital Medical Image software. Surviving neurons were counted in four independent fields of view (×400).

### 2.10 Statistical analysis

Statistical analysis was performed using SPSS statistical software 20.0 (SPSS Inc., Chicago, IL, USA). All data were expressed as mean ± SD. If the comparison between two independent samples meets the normal distribution, the independent sample *t* test is used, otherwise the nonparametric Mann–Whitney *U* test is used. The data were analyzed by one-way analysis of variance if the comparison between three or more samples conforms to normal distribution and has homogeneous variance; the pairwise comparison was performed using the least significant difference (LSD) method. Otherwise the nonparametric Kruskal–Wallis *H* test was used. *P* values <0.05 were considered to be statistically significant.

## 3 Results

### 3.1 Animal survival rate

The survival rate of the 117 rats was 83.76% (98/117), including death after anesthesia (four rats in the ICH group and three rats in the sham-operated group); after modeling, eight rats died before the corresponding time point (three rats died on day 1 and five rats died on day 3); the death time of the experimental animals was usually 1–3 days after the model was prepared, and the death rate was as high as 22%. The rats with Zea Longa score 0 (four rats in the ICH group and one rat in the ICH/HF group) and Zea Longa score 4 (five rats in the ICH group and five rats in the ICH/HF group) were excluded. The success rate of the ICH model was 79.17% (57/72).

### 3.2 Assessment of the ICH model

**Figure 1:**
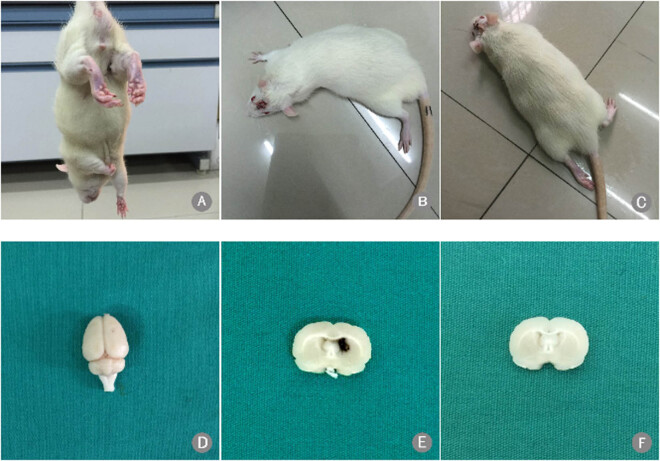
Scoring of intracerebral hemorrhage model and hematoma formation. (A–C) Rats with Zea Longa scores of 1, 2, and 3, respectively; (D) gross brain specimen after autologous blood infusion; (E) hematoma following ICH; and (F) no hematoma in the sham-operated rats.

The ICH model was assessed using Zea Longa scores. A score of 1 indicates that the rat was unable to completely extend its left forelimb (Figure 1A), a score of 2 indicates that the rat circled to the left when walking (Figure 1B), and a score of 3 indicates that the rat fell to the left (Figure 1C). A gross brain specimen of a rat following ICH is shown in Figure 1D. Hematoma was present following ICH (Figure 1E) but not in the sham-operated rats (Figure 1F).

### 3.3 HF treatment reduces serum IL-6 and TNF-α levels in a time-dependent manner

**Figure 2:**
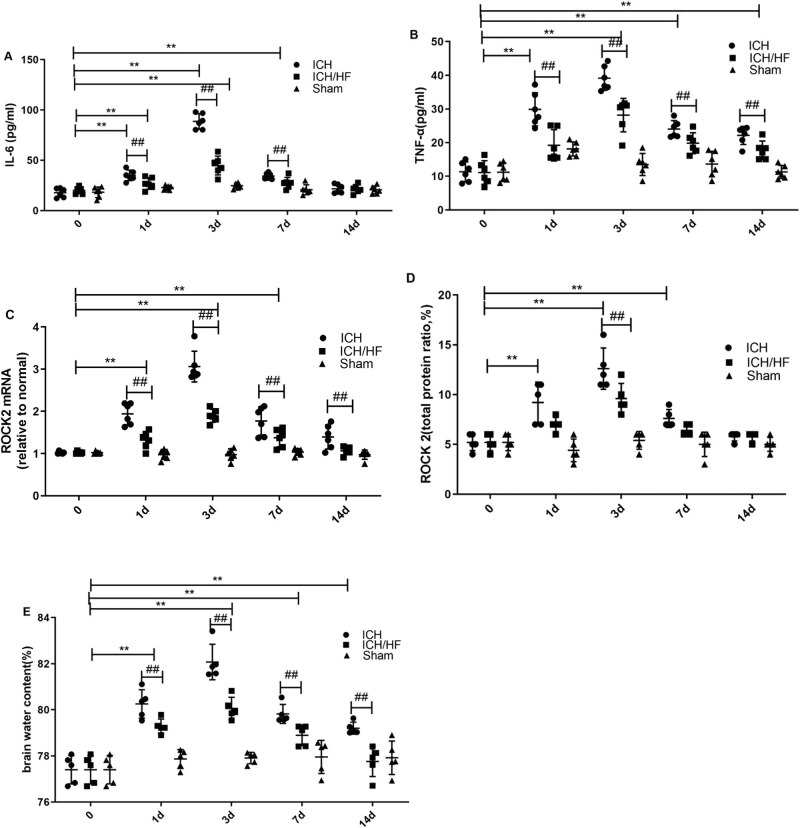
Brain water content and expression of inflammatory factors and ROCK2 on days 1, 3, 7, and 14. (A and B) Levels of serum IL-6 and TNF-α (measured using ELISA, *n* = 6); (C) levels of ROCK2 mRNA (measured using qRT-PCR, *n* = 5); (D) levels of ROCK2 protein in perihematomal area (measured using ELISA, *n* = 5); (E) brain water content (assessed using wet/dry weight method, *n* = 5); data are presented as mean ± SD; **P < 0.05 compared with the normal and sham-operated groups; ^##^ *P* < 0.05 compared with the ICH group.

Serum IL-6 and TNF-α levels were determined by ELISA on days 0, 1, 3, 7, and 14 following ICH. Compared with the normal and sham-operated groups, rats in the ICH group exhibited significantly higher levels of IL-6 and TNF-α (Figure 2A and B, *n* = 6, *P* < 0.05). Treatment with HF significantly decreased the expression of IL-6 and TNF-α at the corresponding time points (Figure 2A and B, *n* = 6, *P* < 0.05). IL-6 and TNF-α levels in the ICH and ICH/HF groups peaked 3 days after the onset of ICH (*P* < 0.05). IL-6 levels in the ICH group at 14 days were not significantly different from those in the ICH/HF and sham-operated groups (*P* > 0.05).

### 3.4 HF treatment inhibits the expression of ROCK2 at different time points

qRT-PCR and ELISA were used to measure the expression of ROCK2 mRNA and protein, respectively, in the perihematomal area. Compared with the normal and sham-operated groups, the levels of ROCK2 mRNA in the ICH group were significantly increased on days 1, 3, and 7 following ICH, peaking on day 3 (Figure 2C, *n* = 5, *P* < 0.05). The expression of ROCK2 mRNA in the ICH/HF group was significantly lower at the corresponding time points (Figure 2C, *n* = 5, *P* < 0.05). The expression of ROCK2 protein essentially aligned with the mRNA expression (Figure 2C and D), except that the expression of ROCK2 protein in the ICH/HF group is not significantly lower than that in the ICH group on days 1 and 7 (Figure 2D, *P* > 0.05).

### 3.5 HF treatment attenuates brain edema from day 1 to day 14 following ICH

Compared with the normal and sham-operated groups, the brain water content was significantly higher in the ICH group at all time points (Figure 2E, *n* = 5, *P* < 0.05). The brain water content in the ICH/HF group was significantly lower than that in the ICH group (Figure 2E, *n* = 5, *P* < 0.05). The brain water content in the ICH group began to increase on day 1 and reached a peak on day 3 (Figure 2E, *n* = 5, *P* < 0.05), suggesting a time-dependent increase in the brain water content following ICH.

### 3.6 HF treatment reduces neutrophil infiltration into the perihematomal areas following ICH

**Figure 3:**
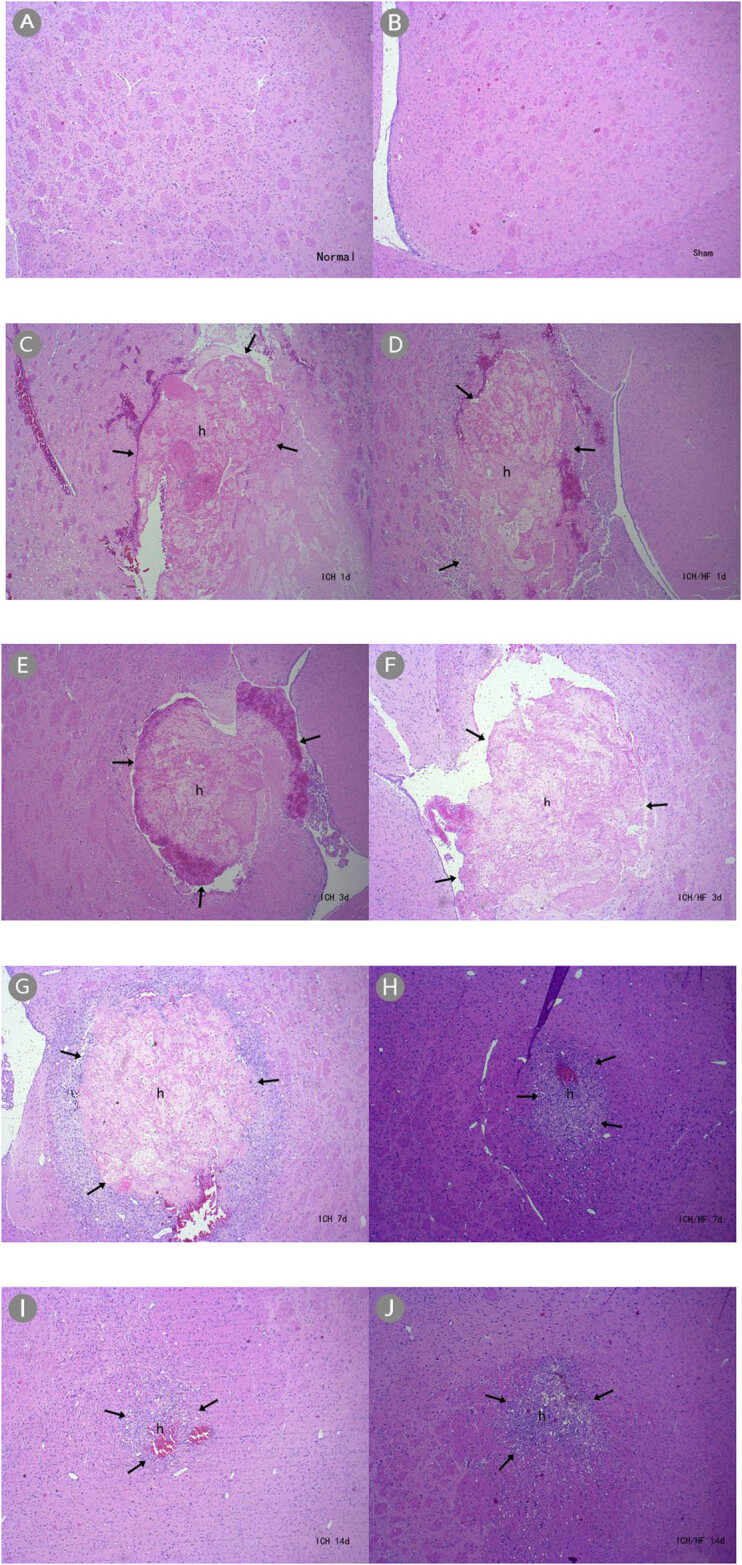
H&E staining showing hematoma formation (×40). (A) Normal and (B) sham-operated groups had normal brain tissue in the basal ganglia; (C–J) ICH and ICH/HF groups had hematomas at each time point.

**Figure 4:**
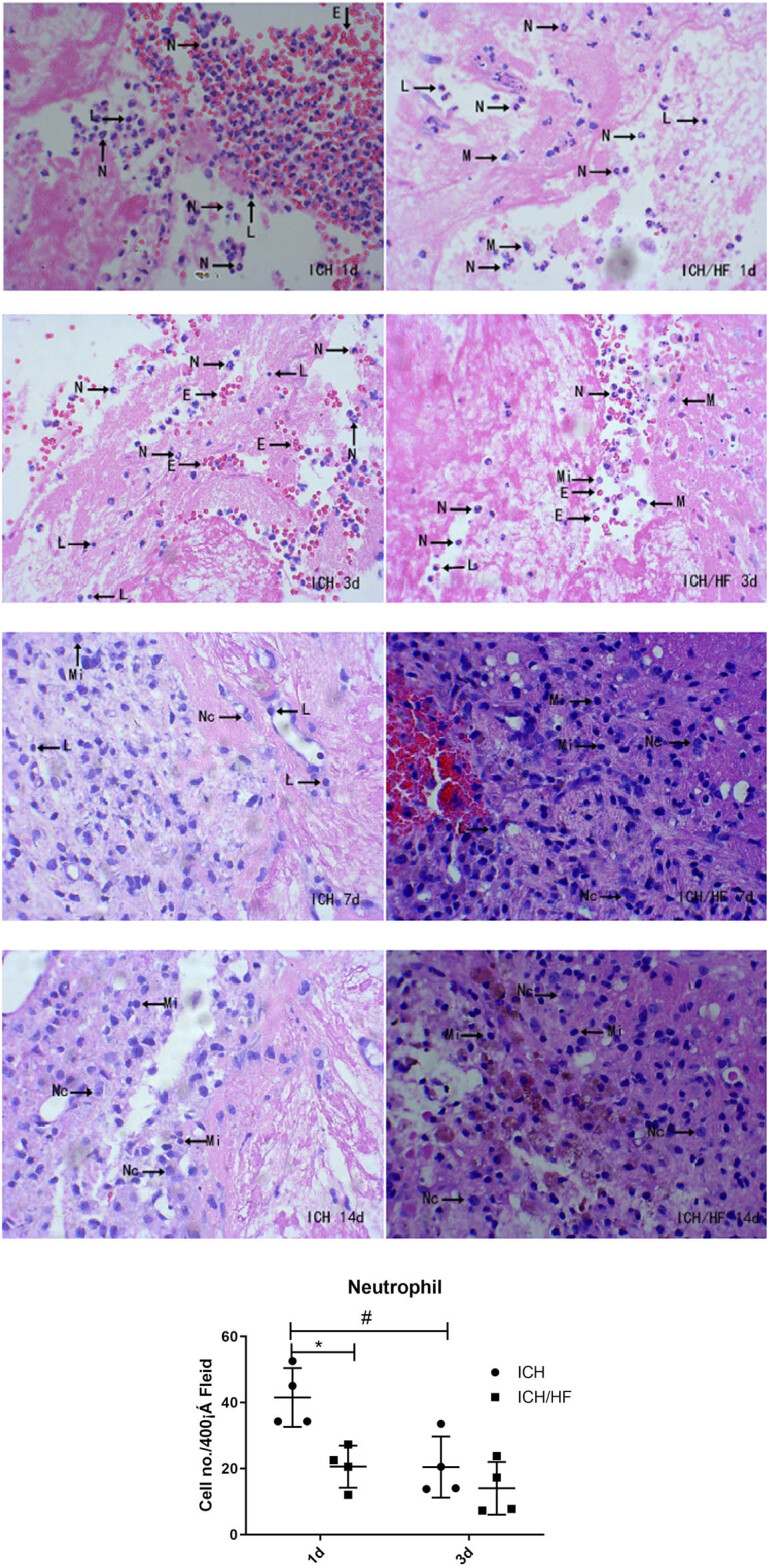
H&E staining of brain sections in the ICH and ICH/HF groups on days 1, 3, 7, and 14 after ICH (×400). Arrows indicate neutrophils (N), lymphocytes (L), macrophages (M), microglia (Mi), neuronal cells (Nc), and erythrocytes (E). In the early stages of ICH (days 1 and 3), a large number of neutrophils were observed in the perihematomal area, with fewer lymphocytes and macrophages. In the later stages of ICH (days 7 and 14), neuronal cells and hemosiderin were present. ^#^ *P* < 0.05 compared with day 1; *P < 0.05 compared with the ICH group (*n* = 4).

H&E staining showed no hematoma formation in rats in the normal (Figure 3A) and sham-operated (Figure 3B) groups, indicating no brain injury. Rats in the ICH and ICH/HF groups, however, showed hematomas at all time points following ICH (Figure 3C–J). H&E staining also revealed inflammatory infiltration around the hematoma in the ICH and ICH/HF groups (Figure 4). At early time points (days 1 and 3), the predominant inflammatory cells in the ICH group were neutrophils, and the number of neutrophils was significantly higher on day 1 than on day 3 (Figure 4, *n* = 4, *P* < 0.05). In comparison with the ICH group, the ICH/HF group showed markedly fewer neutrophils on day 1 after ICH (Figure 4, *n* = 4, *P* < 0.05). A small number of lymphocytes and macrophages were present in the perihematomal area on days 1 and 3.

### 3.7 HF treatment increases neuronal survival following ICH

**Figure 5:**
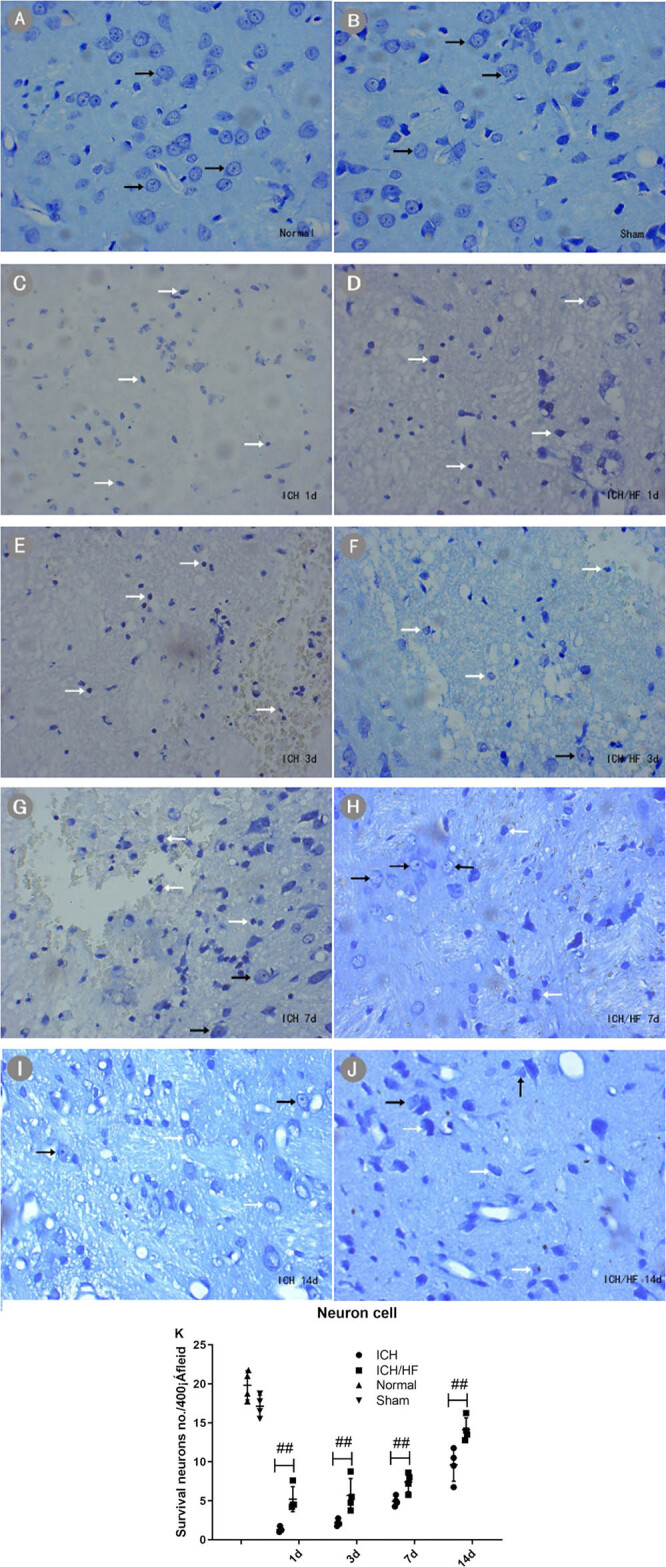
Nissl staining of brain sections from rats in the normal, sham-operated, ICH, and ICH/HF groups (×400). (A) Normal and (B) sham-operated rats had normal neuronal cells (black arrows). (C–J) Damaged neurons (white arrows,) were present in injured brains after the onset of ICH. Damaged neurons were characterized by nuclear pyknosis, nuclear fragmentation, decreased or disappeared Nissl bodies, and cyton swelling, or Nissl staining became shallow and unclear. (K) Quantitative analysis of the number of surviving neurons from ICH and ICH/HF rat brains in the perihematomal area at each time point. Data were collected from four independent fields of view (×400) and are presented as mean ± SD; compared with the ICH group, the ICH/HF group had more surviving neurons at each time point (*n* = 4, ^##^ *P* < 0.05).

An assessment of morphological changes in neurons in the perihematomal area using Nissl staining indicated that neurons of rats in the normal (Figure 5A) and sham-operated (Figure 5B) groups were normal, without any brain damage or change in the neuronal morphology. The injured neurons in the perihematomal area during days 1–14 following ICH in rats were characterized by nuclear pyknosis, nuclear fragmentation, decreased or disappeared Nissl bodies, and cyton swelling, or Nissl staining became shallow and unclear. Nissl staining showed more dead/dying neurons (Figure 5C–J). The quantitative results showed that administration of HF significantly increased the number of surviving neurons in the perihematomal area after ICH induction at all time points (Figure 5K, *n* = 4, *P* < 0.05).

## 4 Discussion

Because of the high sequence identity between ROCK1 and ROCK2 in their kinase domains, it is difficult to design highly specific ROCK1 or ROCK2 inhibitors. Fasudil and Y27632 are widely used nonselective ROCK inhibitors. These inhibitors target ROCK’s ATP-dependent kinase domain and are equipotent in inhibiting both isoforms [17]. Due to these limitations and the nonselectivity of ROCK inhibitors, fasudil is the only ROCK inhibitor approved for human use in Japan and China but not in the United States and Europe [18]. Further research should focus on the development of selective Rho kinase inhibitors, such as ROCK1 inhibitors or ROCK2 inhibitors, to reduce adverse reactions and improve their ability to penetrate the BBB in the treatment of central nervous system diseases. Moreover, since secondary brain injury after stroke is in connection with multifactors, the study of multi-targeted drugs with ROCK inhibition properties will be a focus of these diseases. In this study, we found that fasudil may have a blocking effect on ROCK2, but we did not detect the activity of ROCK2, which is also a limitation of this study. We will further explore this in the future research.

The onset and progression of ICH, especially secondary brain injury after ICH, are complex multifactorial and multilevel pathological processes. Secondary brain injury is associated with inflammation, cytotoxicity, excitotoxicity, disruption of the BBB, and oxidative damage [19]. In this study, using a rat model of ICH, we have shown that HF exerts neuroprotective effects by inhibiting the expression of ROCK2, reducing neutrophil infiltration and inflammatory cytokine levels, decreasing brain edema, and attenuating neuronal loss.

ROCK is involved in the onset and progression of hemorrhagic stroke, and clinical studies have shown that HF promotes the recovery of neurological function after hemorrhagic stroke [20]. Huang et al. showed that ROCK inhibitors can preserve the integrity of the BBB and reduce brain edema and secondary brain injury in a rat model of ICH [21]. Lee et al. showed that HF improved the recovery of neurological function in rats following ICH by activating Wharton’s jelly-derived mesenchymal stromal cells [22]. They suggested that the beneficial effects may involve upregulation of glial cell line-derived neurotrophic factor, which promotes differentiation into neuron-like cells. Our data indicate that the levels of ROCK2 are significantly increased from days 1 to 7 after ICH, peaking at day 3. HF treatment significantly reduced the levels of ROCK2, suggesting that ROCK2 could be closely associated with the injury caused by ICH.

In this study, H&E staining revealed hematomas at all time points following ICH. Other studies have demonstrated the involvement of inflammation in secondary brain injury associated with ICH [23,24]. In our study, H&E staining showed inflammatory cell infiltration in brain tissues around the hematoma. Neutrophils have also been shown to infiltrate the hematoma in the early stages of ICH [25]. Using a rat model of ICH, Gong et al. showed that neutrophil infiltration began in the first 24 h after injury, peaked at day 3 and disappeared between day 3 and day 7 [26]. Chen et al. showed that peak neutrophil-to-lymphocyte ratio (NLR) was associated with the clinical prognosis after severe traumatic brain injury and was a promising predictor for 1 year outcomes [27]. The NLR is also proved to be related to the unfavorable outcomes in ICH [28].

In this study, neutrophil infiltration peaked on day 1 and began to subside on day 3. In agreement with other reports [29,30], we found that neutrophils were rarely present in the later stages of ICH and that microglia and neurons were the predominant cell types. Treatment with HF significantly reduced the neutrophil count on day 1. Like neutrophils, lymphocytes also played a critical role in the inflammatory response. It indicated that T cell lymphocyte played a role in repairing the inflamed tissues [31]. We also found a small quantity of lymphocyte and macrophage infiltration in the perihematomal area following ICH. In agreement with a study by Li et al. [32], we found that serum IL-6 and TNF-α levels began to increase on day 1 and peaked on day 3. After treatment with HF, the levels of IL-6 and TNF-α were significantly decreased, suggesting that neutrophil infiltration precedes expression of serum inflammatory factors. The inflammatory process that follows microglial activation involves infiltration of neutrophils and macrophages, which secrete proinflammatory cytokines, such as IL-6 and TNF-α, and play a critical role in secondary brain injury caused by cerebral ischemia and hypoxia [33,34]. After ICH, TNF-α can trigger the production of other proinflammatory factors, such as IL-6 and IL-1β, leading to inflammation and immune damage [35]. HF decreased the expression of IL-6 and TNF-α and inhibited neutrophil infiltration in rats following ICH

Neutrophils are not always neuroprotective, and they have the potential to break down the BBB, facilitate neuronal cell death, and induce increased expression of oxidative enzymes, all of which will lead to deteriorated outcomes [36]. Neutrophil infiltration in the CNS has been connected with neuronal loss and neurological deficits. In our model of ICH, Nissl staining showed disruption of the normal structure of brain tissue, characterized by vacuolar degeneration, neuronal loss, and cerebral edema. The number of neurons was markedly reduced, or neurons were completely absent, on days 1 and 3 following ICH. Damaged neurons were characterized by karyopyknosis and light cytoplasmic staining. Administration of HF reduced the loss of neurons at all time points following ICH.

Disruption of the BBB after ICH causes cerebral edema, which has a “space-occupying” effect, and leads to the subsequent deterioration, and even death, of patients with ICH. Studies have shown that ICH directly disrupts the BBB, causing increased permeability and increased brain water content [37,38]. ICH-induced activation of ROCK has been shown to lead to phosphorylation of myosin light chains and exacerbation of damage to tight junction proteins, causing early damage to the BBB [39]. ROCK activation can also reduce the stability of the BBB and increase its permeability [40]. Activation of ROCK increases the expression of matrix metalloproteinase 9, which increases the disruption of the BBB and brain edema [39]. In this study, compared with the normal and sham-operated rats, the brain water content in the ICH group was significantly increased, peaking on day 3 after the onset of ICH. This result was consistent with serum IL-6 and TNF-α levels. Treatment with HF significantly reduced the brain water content around the hematoma at all time points, suggesting that cerebral edema may be closely linked to the inflammatory response after ICH. In a rat model of ICH, the ROCK inhibitor HF exerts neuroprotective effects by inhibiting the inflammatory reaction and alleviating brain edema.

### 4.1 Limitations

This study has several limitations. First, in this study, non-anticoagulant autologous arterial blood was injected into the right caudate nucleus to prepare the ICH model, which may be different from the mechanism of spontaneous ICH. Second, the data for hemorrhage volumes at each time point and analyses of differences between groups were not provided in this study. Although the aim of this study was to evaluate the neuroprotective effects of HF in rats with ICH, the activity of Rho-kinase was not measured. Additional research may be required to define the role of Rho-kinase activation in ICH-induced secondary brain injury: for example, measurements of changes in the Rho-kinase activity in the perihematomal brain tissue and blood cells after ICH and determination of the inhibitory effect of HF on Rho-kinase activation. Moreover, we did not study the ICH-triggered apoptosis or neuron necrosis, which is also a limitation of this study. We will further explore this in the future research.

## 5 Conclusion

To our knowledge, ROCK inhibitors have been shown to be helpful for the treatment of several neurological diseases, including spinal cord injury, Alzheimer’s disease, and stroke. However, the effects of ROCK inhibitors in ICH-induced brain injury are poorly reported. Fasudil and Y27632 are the representative nonselective ROCK inhibitors. We demonstrated the protective effects of HF against ICH in the rat model. This study showed that ICH in rats upregulates ROCK2 expression, leading to inflammatory damage, cerebral edema, and loss of neurons. Treatment with HF reduced the expression of ROCK2 and attenuated brain injury following ICH. Although the ROCK signaling pathway in ICH required further study, our results suggested that HF may have the potential for reducing inflammatory injury and alleviating brain edema and subsequent neuronal death in hemorrhagic stroke patients. Our findings indicated that ROCK might be a promising therapeutic target for the treatment of ICH.
